# Biointegration of soft tissue-inspired hydrogels on the chorioallantoic membrane: An experimental characterization

**DOI:** 10.1016/j.mtbio.2025.101508

**Published:** 2025-01-30

**Authors:** Manuel P. Kainz, Mathias Polz, Daniel Ziesel, Marta Nowakowska, Muammer Üçal, Sabine Kienesberger, Sophie Hasiba-Pappas, Raimund Winter, Nassim Ghaffari Tabrizi-Wizsy, Sarah Kager, Theresa Rienmüller, Julia Fuchs, Michele Terzano, Christian Baumgartner, Gerhard A. Holzapfel

**Affiliations:** a Institute of Biomechanics, Graz University of Technology, Austria; b Institute of Health Care Engineering with European Testing Center of Medical Devices, Graz University of Technology, Austria; c Gottfried Schatz Research Center for Cell Signaling, Metabolism and Aging, Division of Medical Physics and Biophysics, Medical University of Graz, Austria; d Department of Neurosurgery, Medical University of Graz, Austria; e BioTechMed-Graz, Austria; f Department of Neurology, Medical University of Graz, Austria; g Institute of Molecular Biosciences, University of Graz, Austria; h Research Unit for Tissue Regeneration, Repair and Reconstruction, Division of Plastic, Aesthetic and Reconstructive Surgery, Department of Surgery, Medical University of Graz, Austria; i Division of Immunology, Research Unit CAM Lab, Otto Loewi Research Center, Medical University of Graz, Austria; j Division of Cell Biology, Histology and Embryology, Gottfried Schatz Research Center, Medical University of Graz, Austria; k Department of Structural Engineering, NTNU, Trondheim, Norway

**Keywords:** Biomimetic hydrogel, Mechanical match, Soft tissue, Tissue engineering, Chorioallantoic membrane, Cell migration

## Abstract

Soft scaffold materials for cell cultures grafted onto the chorioallantoic membrane (CAM) provide innovative solutions for creating physiologically relevant environments by mimicking the host tissue. Biocompatible hydrogels represent an ideal medium for such applications, but the relationship between scaffold mechanical properties and reactions at the biological interface remains poorly understood. This study examines the attachment and integration of soft hydrogels on the CAM using an accessible *ex ovo* system. Composite hydrogels of polyvinyl alcohol and Phytagel were fabricated by sterile freeze-thawing. CAM assays, as an alternative to traditional *in vivo* models, enabled the evaluation of the compatibility, attachment, and biointegration of hydrogels with three distinct compositions. The mechanomimetic properties of the hydrogels were assessed through cyclic compression–tension tests, with nominal peak stresses ranging from 0.26 to 2.82 kPa in tension and −0.33 to −2.92 kPa in compression. Mechanical attachment to the CAM was measured by pull-off tests after five days of incubation. On the first day, the interface strength was similar for all hydrogel compositions. On day 5, softer hydrogels showed the greatest increase (p=0.008), followed by intermediate hydrogels (p=0.020), while the denser hydrogels showed negligible changes (p=0.073). Histological analyses revealed cell infiltration in 100% of soft, 75% of intermediate, and 13% of dense hydrogels, suggesting that softer hydrogels integrate better into the CAM by facilitating cell migration and enhancing interface strength. Chicken embryo survival rates and cytotoxicity assays confirmed the biocompatibility of the hydrogels and supported their potential for use in soft, hydrated three-dimensional scaffolds that mimic tissue environments in dynamic biological systems.

**Statement of significance** Current research on soft scaffold materials for cell cultures often overlooks the critical relationship between mechanical properties and biological integration of these materials with host tissues. Although hydrogels, as soft porous materials, hold promise for creating physiologically relevant environments, the mechanisms driving their attachment and biointegration, especially on the chorioallantoic membrane (CAM), remain largely unexplored. This study addresses this gap by investigating the interaction between soft hydrogels and the CAM, providing valuable insights into how material properties and microstructure influence cellular responses. Our findings emphasize the importance of understanding these dynamics to develop biocompatible scaffolds that better mimic tissue environments, advancing applications in three-dimensional cell cultures on CAM assays and other biological systems.

## Graphical abstract

**Figure:**
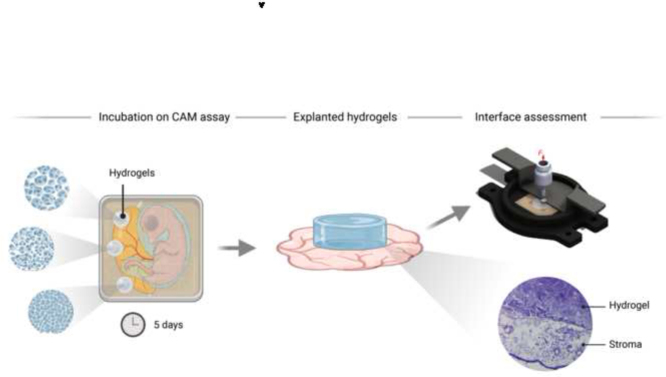

## 1 Introduction

Recent developments in biomaterials for soft-tissue-inspired scaffolds have opened new pathways towards innovative applications in tissue engineering [1], [2], [3], [4], organoid cultures [5], [6], [7], and cell research [8], [9]. By providing a microenvironment closely resembling *in vivo* conditions, tissue-mimicking extracellular matrix materials can support three-dimensional cell growth [10] and provide sophisticated models for cancer research [11], [12], drug delivery [13], and regenerative medicine [14], [15], [16].

**Fig. 1:**
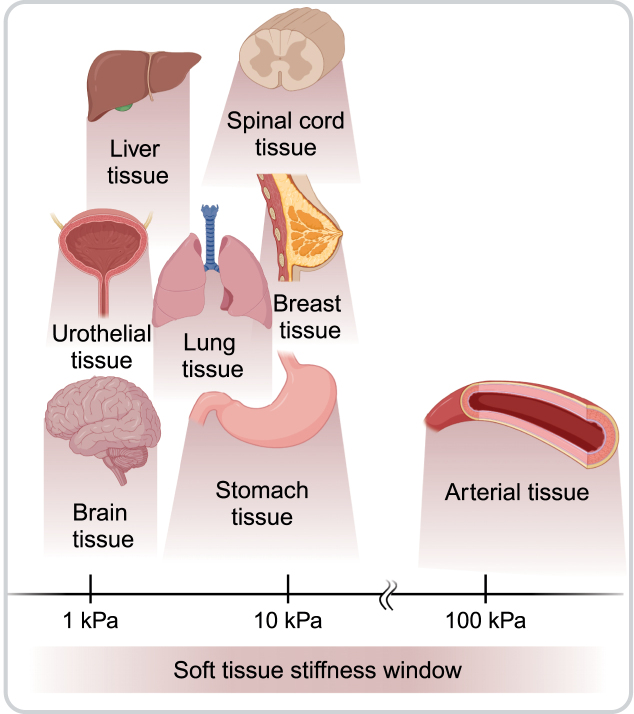
Soft tissue stiffness window: the selection is based on the literature reporting stiffness measures/moduli of healthy, passive, human soft tissues such as brain [55], [56], urothelial [57], liver [58], lung [59], [60], stomach [58], [61], spinal cord [33], [62], breast [63] and arterial tissue [64], [65]. Created with Biorender.com.

Studies show that a morphological and mechanical match between scaffold and host tissue is crucial for the interaction with cells [17], [18], [19], [20]. More specifically, matching of material properties enhances attachment, integration, and improves overall biocompatibility [21], [22], [23]. Among the few suitable materials, hydrogels are able to structurally resemble the tissues’ microenvironment whilst simultaneously exhibiting the viscoelastic mechanical behavior typically observed [24], [25]. Hydrogels have already been applied successfully to mechanically mimic tissues such as lung [26], liver [27], bladder [28], gastrointestinal tract [29], [30], [31], and central nervous system [32], [33], [34], [35], [36]. A schematic of the stiffness window of relevant tissues, which can be potentially covered by mimicking hydrogels, is illustrated in Fig. 1.

Advancements in soft-tissue-inspired scaffolds have made them highly valuable in chorioallantoic membrane (CAM) assays, used to investigate three-dimensional human cancer cell behavior in a dynamic *living* system with higher physiological relevance than *in vitro* experiments [37], [38], [39]. The CAM assay is a widely used model offering a cost-effective platform to study tumor development [40], [41], [42], drug treatment [43], biomaterials [44], [45], and angiogenic and inflammatory processes [46], [47], [48]. The accessibility of the CAM model makes it invaluable for preclinical studies, whilst avoiding pain and suffering in line with the ‘Three Rs’ principle [49], [50]. As a matter of fact, experiments can be conducted without ethical restrictions or the need for prior approvals until day 14 of embryonic development [51]. The CAM assay provides valuable preliminary insights, particularly in the early stages of materials testing, while traditional animal models enable a more comprehensive understanding of the hydrogel performance, including immune responses and long-term risks. Therefore, both models are complementary, with the CAM assay providing valuable preliminary insights, while traditional animal models, including rodents, are still needed for a more comprehensive understanding of hydrogel performance and potential risks [52]. In CAM assays for cell studies, both the interactions between grafted cells and the scaffold, as well as the interactions between the scaffold and the CAM membrane, are critical in determining how effectively the scaffold integrates and functions within the system. A key aspect of this is biointegration, which plays a vital role in optimizing scaffold design for *in vivo* applications. Biointegration is the process by which a biomaterial becomes fully incorporated into surrounding tissue. It involves not only physical attachment but also cell growth and tissue formation around and through the material, creating a stable, functional bond. Unlike simple attachment, biointegration promotes cellular migration, proliferation, and differentiation, ensuring a seamless, biocompatible connection with the host tissue [53], [54].

Despite its fundamental role in the interaction between soft hydrogels and host tissue, the interface behavior and the corresponding cellular reactions have been given little attention so far. In CAM experiments, studying the interface with the membrane is essential and can have implications for cell viability, nutrient supply and the overall successful use of the CAM model for preclinical assessments. Although hydrogel–CAM interfaces have been extensively examined by histological means, a mechanical assessment is missing; specifically, it remains unclear how the material properties of the hydrogel influence its integration with the biological membrane. A collection of studies that have previously explored hydrogels in combination with CAM assays is provided in the Supplementary Material.

In this work, we characterize a soft tissue-mimicking hydrogel and investigate its usability and integrability with the chicken embryo CAM. The hydrogel, a composite of polyvinyl alcohol (PVA) and Phytagel (PHY), is of particular interest due to its mechanical similarities to soft hydrated biological tissues [66], as well as its demonstrated biocompatibility with cells [67]. Introduced as a brain phantom material [68], this composite hydrogel has shown excellent mechanomimetic abilities, particularly in replicating the viscoelastic behavior of brain tissue [69], [70]. The unique combination of mechanical match, tunability, biocompatibility of the constituents [71], [72], and porous microstructure suggest that this material is well-suited for soft-tissue-inspired scaffolds and can provide a highly physiologically relevant microenvironment to facilitate cellular integration on the CAM. To expand the investigation beyond the established brain-mimicking material, we additionally investigated formulations with lower and higher polymer concentrations, as previously presented by Tan et al. [66]. This approach allows for a more comprehensive assessment of the mechanical behavior and biocompatibility of the hydrogel across a range of compositions. An established method for fabricating composite hydrogels [68], [70] was here adapted to allow for sterile material synthesis. Mechanical match of the biomaterial to soft tissues was qualitatively and quantitatively assessed with compression–tension experiments. The microstructure was examined using cryo scanning electron microscopy. To validate the sterility and cell compatibility, maturing neurons were used as a high-sensitivity model. Hydrogels with varying polymer fractions were grafted and incubated. After several days of direct contact to the CAM, mechanical pull-off tests were conducted to quantify attachment, and combined with an examination of the interface microstructure to assess integration. This setup, applied here for the first time in the context of scaffold–CAM interactions, represents a novel approach to studying mechanical integration at this interface. Histological examinations of cryosectioned hydrogels discs followed, providing further insights into cellular responses and potential effects on the membrane.

This study addresses the critical challenge of understanding the interface between scaffold materials and biological tissues, with particular emphasis on the CAM model as a representative soft tissue environment. Studying this interaction provides new insights into how soft tissue-mimicking hydrogels attach to and integrate with biological membranes. This closes a gap in previous research that largely ignored the mechanical and biological integration aspects at this interface. These findings could not only improve scaffold design for soft tissue engineering, but also contribute to the development of hydrogels optimized for various biomedical applications, including regenerative medicine, organoid development, and drug delivery systems. In contrast to previous studies that comprehensively investigated composite hydrogels, this work focuses exclusively on the mechanical assessment and integration behavior of hydrogels in the CAM model and provides a basis for further development of preclinical testing of biomaterials.

## 2 Materials and methods

### 2.1 Composite hydrogels

#### 2.1.1 Sterile hydrogel fabrication

**Fig. 2:**
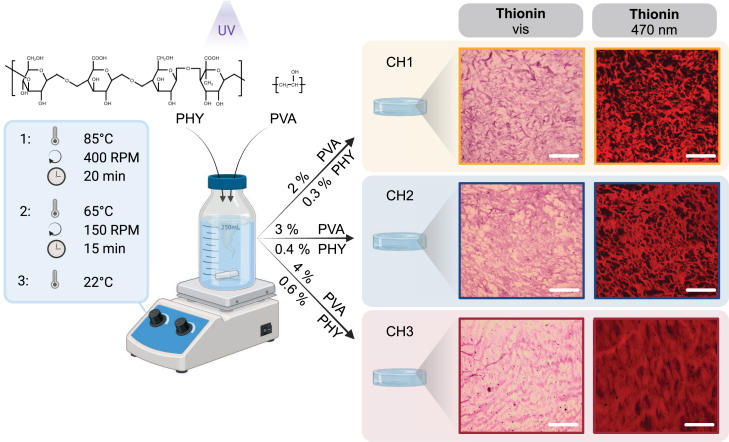
Sterile PVA-PHY composite hydrogel (CH) fabrication and morphological assessment. Sketch of the fabrication under UV-light, applied to three different compositions with varying concentrations (w/w%). The structural formulas show the polymer structures of PVA and PHY. Morphological images of each composition were obtained using Thionin-stained cryosectioned slices in bright field (vis) and fluorescence microscopy excited at 470 nm. Scale bars indicate 200 μm. Created with Biorender.com.

Hydrogel fabrication (Fig. 2) is based on previous studies using a freeze-thaw technique, improved to facilitate reproducibility in a highly controlled environment [68], [70]. To ensure uniform heat distribution, the beakers were wrapped with aluminum foil and sealed with screw-on lids. Rod thermometers with thermostats for temperature control were inserted through a hole in the lid to avoid water vapor from escaping. As a result, no additional liquid needed to be replenished during or after the heating process. Three different types of composite hydrogels (CH) with varying concentrations (w/w%) of PVA and PHY were fabricated: CH1: 2% PVA, 0.3% PHY; CH2: 3% PVA, 0.4% PHY; and CH3: 4% PVA, 0.6% PHY. PVA (Sigma Aldrich, Mw 146.000−186.000, 99+% hydrolyzed) and PHY (Sigma Aldrich) were directly mixed with deionized water and then heated to 85°C at 400  rpm for 20  min instead of being heated separately as proposed in the mentioned studies [68], [70]. The mixture was then cooled to 65°C at 150  rpm for 15  min. The solution was filled into beakers (17  cm diameter, 120  cm height) and put to rest for 2  h at room temperature (22°C) with the lid open. Next, cryogels were produced by freezing the polymer solution at −20°C for 24  h, thereby forming ice crystals that act as pore-shaping agents. In a last step, the tubes were thawed at 22°C for 4  h resulting in the composite hydrogels used for this study. Precursor materials, PVA-PHY mix and the resulting hydrogel were sterilized in the corresponding production step for 15  min under UV light. The entire fabrication process was carried out in a laminar flow hood to minimize contamination and ensure a stable temperature. The permeation of UV radiation was tested with UV absorption spectra, and microbial contamination tests were employed as a quality control for each fabrication batch to ensure the purity of the fabricated material (Supplementary Material). Unless specifically mentioned, the hydrogel samples were stored in deionized water to prevent dehydration and to ensure a fully fluid-saturated material.

#### 2.1.2 Colorimetric cytotoxicity assay

Cytotoxicity of precursor materials was evaluated in previous studies [71], [72], but to the best of the authors’ knowledge it was not assessed for the interaction of cortical neurons and the proposed composite hydrogel before. Therefore, cytotoxicity was evaluated on primary cortical neurons of neonatal (P0-P1) rats in an indirect contact colorimetric lactic dehydrogenase (LDH) assay according to the standard DIN EN ISO 10993-5 for the biological evaluation of medical devices. A preliminary study (n=6) was conducted to determine the testing frequency, followed by the main experiment with n=12 tests. Half of the wells were used as negative control group. This comprehensive approach ensured a robust evaluation of the interaction of the hydrogel with neurons and its safe use in follow-up experiments in *in vivo* models. Rat pups for primary cell cultures were obtained from the in-house breeding of Sprague Dawley rats, which were housed in the animal facility of the Biomedical Research Institute at the Medical University of Graz and maintained at a constant room temperature of 21±1°C, relative humidity of 50±5 %, and a 12  h light/dark cycle. Pups were sacrificed on the first day after birth, the skull was opened, and the brain was carefully removed and placed in ice-cold, sterile phosphate-buffered saline (PBS) (pH 7.4). All non-cortical brain parts were removed and the cortex was finely dissected using a tissue chopper. The chopped tissue was then enzymatically digested with Accutase at 37°C for 20  min. Cells were then seeded into poly-D-lysine (0.1  mg/ml) coated 96-well plates. The seeding density reached 16000  cells/sample (50000  cells/cm^2^). The sample size for each group was n=6. The LDH test was performed in triplicate. Culture media (Neurobasal A, B27 2%, GlutaMAX 125  mM, bFGF 5  ng/mL, Normocin) were preconditioned by contact with a CH2 hydrogel disc (4  mm diameter, 2  mm height) for one day. LDH levels were measured in supernatants of cells exposed to preconditioned media 48  h after cell attachment to the well plate. The LDH assay was performed utilizing a commercial LDH Cytotoxicity Assay kit (Invitrogen CyQUANT) according to manufacturer’s protocol. To set the baseline for LDH activity measurements, an extra sample from the control group underwent lysis to establish the maximum LDH activity. Absorbance readings were obtained using a microplate reader (SPECTROstar Omega). Cytotoxicity levels were determined as a percentage of LDH activity relative to the established maximum LDH release control (*I*${}_{\max}$), as outlined in the product data sheet. Cytotoxicity was calculated and corrected by the absorbance of the control in water using the formula

(1) $$\text{Cytotoxicity}\;\left[\text{\%}\right]=\frac{\Delta I_{\mathrm{CH}}-\Delta I_{\mathrm{Control}}}{I_{\max}-\Delta I_{\mathrm{Control}}}\cdot 100\;,$$

where $\Delta I_{\mathrm{CH}}$ and $\Delta I_{\mathrm{Control}}$ are the differences of the absorbance values of the sample at 490  nm and 680  nm for the supernatant and the water control, respectively. This calculation measures the normalized difference in absorbance between viable cells and background absorbance: higher absorbance differences indicate greater cytotoxicity, as they reflect a larger reduction in cell viability compared to the control group.

#### 2.1.3 Image acquisition and microstructure investigation

Slices 5μm thick were cut along the transversal plane from the prepared hydrogel discs using a microtome (Epredia CryoStar, NX50 Kryostat) while keeping them at −20°C. Slices were then transferred onto SuperFrost Plus™ slides (Thermo Fisher Scientific). This procedure allows for a two-dimensional estimation of the microstructure (Fig. 2).

Under examination using bright field (Olympus BX51) and phase contrast microscopy (Olympus CKX53), the hydrogel appeared to be highly transparent. Post-processing of the images by enhancing contrast and saturation improved the visibility of the hydrogel, but did not accurately represent the network morphology. Therefore, a fluorescence microscope (Olympus BX51 equipped with CoolLED pE-300 LED Illumination System) was used to visualize the internal morphology. The excitation filter used was a band pass filter at 470–490  nm, the emission filter was a low pass filter at 520 nm, and a dichromatic mirror with a cut-off at 500  nm was utilized. Influence of structural changes, such as swelling or dissolution of the hydrogel during the staining process, were found to be negligible based on piloting tests under a bright field microscope. Thionin (Sigma Aldrich, St. Louis, United States) was selected due to the common use of the dye in fluorescence microscopy and the high contrast of Thionin-stained tissue in bright-field microscopy. The agent is fluorescent at a wavelength of 470  nm−490  nm [73], [74]. A non-alcoholic protocol for both staining agents was established. A drop of 100  μl of Thionin was added on top of the 5  μm slides. After 5  min, the slices were washed with distilled water and then left to dry for 10  min. No mounting medium was applied for preservation.

Additionally, the microstructure of the composite hydrogels was investigated using cryo scanning electron microscopy (cryo-SEM). Cylindrical samples were frozen in slush liquid nitrogen and transferred under vacuum to a cryo preparation chamber connected to a GEMINI Sigma 500 SEM (Zeiss Group, Oberkochen, Germany). The samples were fractured, sublimated, and sputter-coated with palladium. Micrographs were taken using a Sigma 500VP FE-SEM with a secondary-electron detector operated at an acceleration voltage of 5  kV under vacuum conditions to maintain sample integrity. To analyze the polymer network structure and distinguish it from the background, the open-source software ImageJ [75] was used. The enhanced contrast between the polymer network and the frozen background enabled clear visualization of the network features. The structure was converted to a binary image using black-and-white threshold filters, effectively isolating the polymer network. The *Analyze Particles* tool in ImageJ was then used to identify the pores, allowing detailed characterization of the microstructure of the hydrogel composites.

#### 2.1.4 Mechanical characterization

Cyclic uniaxial compression–tension experiments were conducted to quantify and compare the mechanical behavior of the three different hydrogel composites and to assess their mechanomimetic nature with respect to soft biological tissues. The mechanical tests were performed with a triaxial testing device previously introduced [76]. Discs were prepared from synthesized bulk material, trimmed to a height of h=6±2  mm, and then cut with a commercial biopsy punch to a nominal diameter of d=10±1  mm. Throughout preparation and testing, the samples were kept hydrated with deionized water. The samples were attached to parallel sample holders using sandpaper, superglue, and double-sided tape. During the initialization, the upper part with the glued samples moved towards the lower part with a speed of v=3  mm/min. The samples were pushed toward the sample holder until a preload of 5  mN was reached. The position was maintained for 2  min for the glue to dry, followed by a relaxation period of 5  min. Next, cyclic compression–tension loading with 15% vertical strain was applied at a constant stretch rate of $\overset{˙}{\lambda }=0.005$  s^−1^. The loading speed v was adjusted based on the initial sample height h with $v=\overset{˙}{\lambda }h$. The vertical displacement Δh measured during each test was used to derive the stretch ratio λ=(h+Δh)/h, with maximum λ=0.85 and λ=1.15 for compressive and tensile loading, respectively. Vertical forces and displacements were recorded over time. The nominal stress was derived by dividing the vertical forces by the initial cross-sectional area of each sample. Each test began with compressive loading and included four cycles. In total, n=10 samples of CH1, n=6 samples of CH2, and n=10 samples of CH3 were included in this work. Unless otherwise stated, all mechanical experiments were performed at a temperature of 22±2°C.

### 2.2 Hydrogel–tissue interactions in the chick embryo model

#### 2.2.1 Hydrogels on CAM

**Fig. 3:**
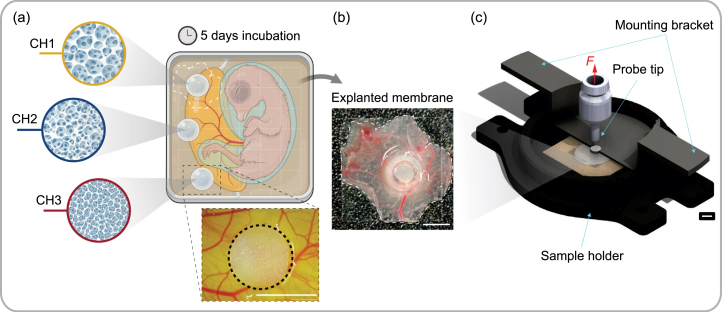
Experimental assessment of mechanical integration of hydrogel samples on CAM. (a) Illustration of a CAM assay with samples from three distinct hydrogel compositions CH1, CH2 and CH3. The inset shows a sample on day 1; (b) explanted membrane with hydrogel disc on a microscopy slide 5 days after application; (c) uniaxial pull-off test setup and probe tip, where *F* indicates the force needed to pull the hydrogel sample from the membrane. Scale bars indicate 5 mm. Created with Biorender.com.

Cell–hydrogel interactions were tested using CAM assays as an alternative to *in vivo* experiments with pain receptive animals. *Ex ovo* CAM assays were performed according to Deryugina and Quigley using fertilized white leghorn chicken eggs [77]. The detailed procedure can be found in the Supplementary Material. Hydrogel discs were prepared from sterilized bulk material, cut in slices of h=2  mm using a spring steel blade, and then punched with a commercial biopsy punch with 5  mm in diameter. Throughout preparation, the hydrogel samples were kept hydrated with deionized water. Samples were carefully grafted between embryonic blood vessels on the CAM on day 10 of embryonic development and fixed in place with silicone rings, 0.5  mm in height and 5  mm in inner diameter, for better localization of the sample because of the highly transparent nature of the hydrogels [78]. As illustrated in Fig. 3(a), one disc of each hydrogel composition CH1, CH2 and CH3 was applied to the chick embryos on top of the CAM, ensuring that the reaction to the biomaterial is not dependent on one particular host organism. A disc of semipermeable wax-based film (Parafilm, Bemis Company, Inc., Neenah, USA) was placed on top of each sample to allow gas exchange with the environment while reducing sample drying. The film was specifically selected for its semi-permeable nature, ease of handling and ability to maintain a controlled moisture level around the sample, effectively reducing the risk of drying during incubation. After application, the embryos were incubated for five more days. The hydrogels were photographed daily and harvested with the surrounding membrane via excision after 5 days of incubation. After harvesting, the samples were either directly transferred onto a glass coverslip for the mechanical pull-off test (Section 2.2.2) or shock frozen and stored at −80°C for histological assessments (Section 2.2.3).

#### 2.2.2 Uniaxial pull-off test and interface strength

Pull-off tests were conducted to evaluate the mechanical attachment of hydrogel discs to the biological membrane using a modified triaxial loading device (see Section 2.1.4). The setup included a custom sample holder with a precise clamping system for soft, membrane-like materials, consisting of a lower sample stage, a mounting bracket for central alignment, and a modified probe tip connected to the load cell (Fig. 3(c)). The probe tip, a flat punch with a diameter of 4  mm, was equipped with sandpaper to enhance contact with the hydrogel sample. Hydrogel samples were excised with the surrounding membrane on day 5 after incubation and placed on glass coverslips (18 × 18  mm), as described in Section 2.2.1. An example is shown in Fig. 3(b). Tests were performed on n=24 samples with n=8 of each composition, from n=8 different CAM assays. Out of the n=24 samples, n=15 were included for post-processing based on drop-out criteria of insufficient hydrogel-probe attachment or misalignment causing gluing to the silicone ring. Benchmark tests on day 1 involved n=3 samples of each hydrogel composition from n=3 different CAM assays, of which n=8 could be used for post-processing. Membrane hydration was ensured by removing samples right before testing, and each test was completed within 1  h post-excision.

The displacement-controlled testing protocol started with positioning the probe tip well above the sample to apply an acrylate-based adhesive to the hydrogel with a syringe. During the contact procedure, the probe tip was lowered until a reaction force of 2  mN was achieved and maintained for 2  min to allow the adhesive to set, based on pre-established drying times. The probe was then retracted to a zero-force reading and then lifted with a constant speed of 1  mm/min until complete detachment, indicated visually and by a stable force reading representing the weight of the hydrogel sample. Sample diameters were measured with a digital caliper, and close-up images were taken after each test.

The experimental results were obtained in the form of time-resolved force–displacement data. As a measure for the attachment between hydrogel and CAM, the interface strength was computed based on the force–displacement data of each sample. We defined the interface strength as the energy per unit area required to fully detach the material from the membrane. This can be formalized by

(2) $$\Gamma =\frac{1}{A}\int_{z=0}^{z=\delta_{c}}Fdz\;,$$

where A is the contact area between sample and membrane, F is the tensile force in vertical direction in the integral from the starting point z=0 to the point of complete detachment $z=\delta_{c}$. The interface strength was derived by numerically integrating the force–displacement curve from zero to the point of constant force (complete detachment) and dividing by the contact area of the hydrogel on the membrane. Force data were corrected by removing the corresponding weight of each sample.

#### 2.2.3 Cryosectioning and histology

Following standard protocols, the explant consisting of the hydrogel and surrounding CAM was fixed in 4% paraformaldehyde for 24  h [79] and then washed in 1xPBS, placed with the CAM side on aluminum foil. The silicone ring was carefully removed using a tweezer. The surrounding liquid was then absorbed with precision wipes. Aluminum foil and sample were placed on a metal block of dry ice to freeze the explant. The frozen explants were then embedded in Tissue-Tek® O.C.T. Compound at −22°C, sectioned at the center along the sagittal plane to 5μm slices using a cryotome (Epredia CryoStar, NX50 Kryostat), mounted on SuperFrost Plus™ slides (Thermo Fisher Scientific) and air-dried for 24  hours.

The cryosections were stained using a non-alcoholic protocol for Hematoxylin and Eosin staining. In short, slides were placed in Hematoxylin for 1  min 45  s, washed briefly in distilled water, rinsed for 3  min under warm tap water to blue the Hematoxylin and washed again in distilled water. Subsequently, the sections were stained with 0.2% Eosin for 30  s, washed in distilled water and finally covered with mounting medium (AquaTex, Merch). In total, n=24 samples were explanted and cryosectioned three times, resulting in n=72 successful cryosections. The most successful of three cryosections per hydrogel sample was used for further analysis.

### 2.3 Statistical analyses

Statistical analysis of the data from the cytotoxicity assay was performed using R Statistical Software (v4.2.0, 2022-04-22) for Mann–Whitney U tests [80]. A Fisher’s exact test was used to compare the survival rates of chicken embryos subjected to the material with those in a negative control group. Statistical analysis of the data from the pull-off test was performed using Welch’s unequal variances t-test to compare the means of CH1, CH2, and CH3, accounting for unequal sample sizes and variances. Pairwise comparisons (CH1 vs. CH2, CH1 vs. CH3, CH2 vs. CH3) were conducted. All analyses were considered statistically significant for p-value < 0.05.

## 3 Results and discussion

For clarity, this section presents the discussion together with the results. Qualitatively, we assessed the impact of hydrogel microstructure and mechanical behavior on biointegration, emphasizing trends in cell infiltration and material integration. We quantitatively assessed biocompatibility through cytotoxicity assays and embryonic survival rates, alongside evaluating mechanical behavior and stiffness using peak stresses derived from cyclic compression–tension tests. Additionally, the interface strength from pull-off tests on hydrogel–CAM interface was compared using statistical analyses.

### 3.1 Biocompatibility assessment

#### 3.1.1 High sensitivity model shows no cytotoxic effects of the hydrogel on maturing neurons

Primary cortical neurons were used as a precautionary measure due to their high sensitivity, which makes them a suitable model for examining short term effects on maturing cells. The use of a sensitive cell type ensures that subtle cytotoxic effects are detected, which may remain unnoticed when using less sensitive cell types. The results of the indirect contact test with media preconditioned with a hydrogel on day *in vitro* 7 (DIV7), 48  h after incubation, revealed no significant cytotoxicity towards maturing neurons compared to the negative control group without contact (Supplementary Material). Notably, the cytotoxicity percentage, Eq. (1), between the hydrogel-conditioned media (n=6, median: 25.32) and the control group (n=6, median: 19.15) did not show statistically significant differences (W=9, p=0.18).

This suggests that the presence of the hydrogel may not induce nor mitigate cytotoxic effects on neurons, leaving space for further investigation in *in vivo* experiments. In addition to comparing cytotoxicity percentages between the hydrogel-conditioned media and the control group, further analyses could explore additional parameters such as cell viability, morphology, and functionality to gain a more comprehensive understanding of the biocompatibility of the hydrogel. However, evaluating cytotoxicity in maturing neurons already provides crucial preliminary data regarding the suitability of the material for further *in vivo* model studies.

#### 3.1.2 Hydrogels have no influence on embryo survival rate

The CAM assay can be used not only to study the interaction on the membrane, but also to investigate the biocompatibility of biomaterials in direct contact. A total of n=44 *ex ovo* CAM assays in contact with the hydrogels showed an overall survival rate of 68.2% (n=30). In contrast, a negative control group with n=24 CAM assays showed a similar survival rate of 70.8% (n=17). The quantitative comparison yielded a p-value of 1.0, indicating no statistically significant difference in survival rates between the two groups (see Supplementary Material). These results indicate that the presence of the hydrogel on the CAM did not have a significantly adverse impact on the mortality.

### 3.2 Biomechanical characterization

#### 3.2.1 Mechanomimetic behavior of hydrogels can be tuned by varying the polymer concentration

**Fig. 4:**
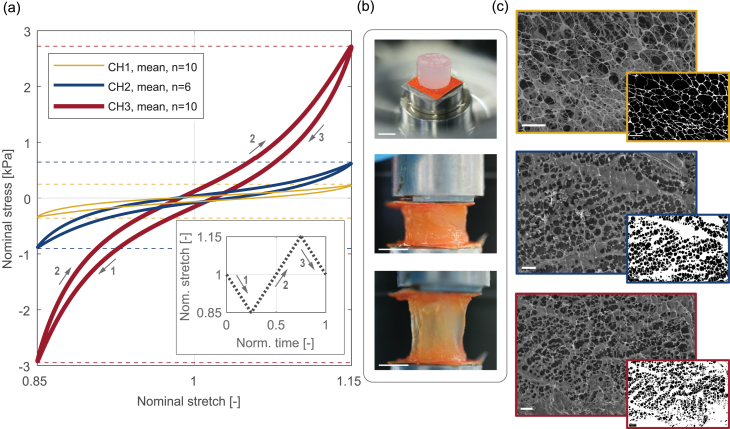
Compression–tension behavior of composite hydrogels. (a) Mean uniaxial stress–stretch curves (last of four cycles). Peak stresses are indicated with dashed lines. The inset shows a complete loading cycle, where step 1 indicates compression to λ=0.85, step 2 indicates tension to λ=1.15 and step 3 indicates unloading back to λ=1; (b) example images of a hydrogel before testing (top), during compression (middle), and during tension (bottom). Scale bars indicate 5 mm; (c) microstructure of hydrogels CH1 (top), CH2 (middle) and CH3 (bottom) from cryo-SEM. The insets show binary images after removing the background features. Scale bars indicate 10 μm.

The macroscopic mechanical behavior of the three different hydrogel compositions, assessed from cyclic uniaxial compression–tension experiments, is reported in Fig. 4(a), which shows the mean curves obtained from the last of four loading–unloading cycles. Fig. 4(b) shows representative images of a sample before testing (top), during compression (center) and during tension (bottom). It appears that, by varying the polymer concentration, the mechanical behavior of the hydrogels can be adjusted and tuned, thereby covering a good part of the soft tissue stiffness window (see Fig. 1). These results provide consistent data with previous studies (in the CH2 composition) [66], [68]. Notably, the mechanical behavior of the CH1 and CH3 compositions has not yet been characterized and quantified, and this study provides important data that positions these hydrogels, with their varying polymer concentrations, within the soft tissue window.

As shown in Fig. 4(a), the response of the hydrogels is highly nonlinear with a distinct compression–tension asymmetry present in the mean data of all three composites. The nominal stress peak values in compression, corresponding to a stretch of λ=0.85, are ${\overset{ˆ}{P}}_{{\mathrm{CH1}}^{-}}=-0.33$  kPa, ${\overset{ˆ}{P}}_{{\mathrm{CH2}}^{-}}=-0.88$  kPa and ${\overset{ˆ}{P}}_{{\mathrm{CH3}}^{-}}=-2.92$  kPa, whereas in tension, at a stretch of λ=1.15, they are ${\overset{ˆ}{P}}_{{\mathrm{CH1}}^{+}}=0.26$  kPa, ${\overset{ˆ}{P}}_{{\mathrm{CH2}}^{+}}=0.62$  kPa and ${\overset{ˆ}{P}}_{{\mathrm{CH3}}^{+}}=2.82$  kPa. Comparing the different compositions, in CH3 the (absolute) peak stress is 8% higher in compression than in tension. In the case of CH1, the difference is 29%. Particularly, this composition can represent a lower limit, marking the transition from a viscoelastic soft solid to a fluid-like material. With 42%, the difference between compressive and tensile peak stresses is most prominent for composite CH2, which is the composition originally designed as a brain tissue-mimicking material [68]. Overall, the behavior observed appears to be consistent with experimental observations in human brain tissue, although the compression–tension asymmetry is less pronounced in the hydrogel. The reported stresses agree well with the behavior of brain tissue when subjected to similar strain rates on the order of $O\left(10^{-3}\right)$  s^−1^ to $O\left(10^{-4}\right)$  s^−1^ and under similar applied strains [55], [81], [82].

More in general, the results obtained underline the mechanomimetic character of the PVA-PHY composite hydrogels. Notably, the hydrogel is able to qualitatively reproduce various mechanical features of soft biological tissues, including nonlinearity, compression–tension asymmetry and loading–unloading hysteresis [83], as reported in various biomaterials [66], [84], [85], [86], [87], [88]. The effect of conditioning and hysteresis during the compression–tension tests are further discussed in the Supplementary Material. As stated in Section 2.1.1, we used an established method for fabricating composite hydrogels [68], [70], adapted here to enable sterile material synthesis, consisting of just one freeze-thaw cycle. Increasing the number of freeze-thaw cycles is expected to alter stiffness, elasticity and stability of the PVA-based hydrogels due to enhanced phase separation and crystallization [89]. Future studies could investigate the effects of different freeze-thaw cycles, temperature gradients, and freezing/thawing durations to further optimize the mechanical properties of hydrogels for specific soft tissue applications.

#### 3.2.2 Relationship between network density and polymer concentration confirmed across hydrogels CH1 to CH3

While the mechanical match with soft biological tissues is relevant, the biomimetic capability of a synthetic material extends beyond its mechanical properties and cannot be reduced to a single parameter (e.g., tissue stiffness or peak stress). In other words, a biomechanical assessment is necessary but not sufficient to soundly support the applicability of a biomaterial for biomedical use [53], [54].

Therefore, we also analyzed the microstructural morphology of the three hydrogels, which was clearly observable with the Thionin staining in the visible range as well as under fluorescence (Fig. 2). Enlarged views are shown in the Supplementary Material. While the bright field observations of Thionin-stained hydrogels provided a general overview, Thionin illuminated with a wavelength of 470  nm revealed distinct features at the microscale. The contrast to the black background particularly highlights the pronounced porous structure. The observed pore distribution was mostly homogeneous, with a trend of decreasing pore size from CH1 towards CH3. Qualitatively, CH1 and CH2 exhibit similar surface porosity, whereas CH3 presents a notably denser microstructure, confirming earlier correlations between pore size and polymer fraction [68], [70]. This also indicates that the modifications to the fabrication method, particularly the UV treatment, does not negatively affect the composite hydrogel morphology and controllability thereof.

A more detailed analysis of the SEM micrographs reveals a distinct microstructure of the three hydrogel compositions, reflected in their mechanical properties. As shown in Fig. 4(c), the softest hydrogel CH1 has a coarse polymer network with large, well-defined pores about 10  μm in diameter, while the denser hydrogels have smaller pores (5-10  μm in CH2 and below 5  μm in CH3) and increasingly compact structures. The progressive reduction in pore fraction at higher polymer concentrations correlates well with the observed increase in mechanical stiffness and shows a clear relationship between polymer content, microstructure, and mechanical behavior.

#### 3.2.3 Qualitative assessment shows distinct attachment of hydrogel on the membrane after incubation

**Fig. 5:**
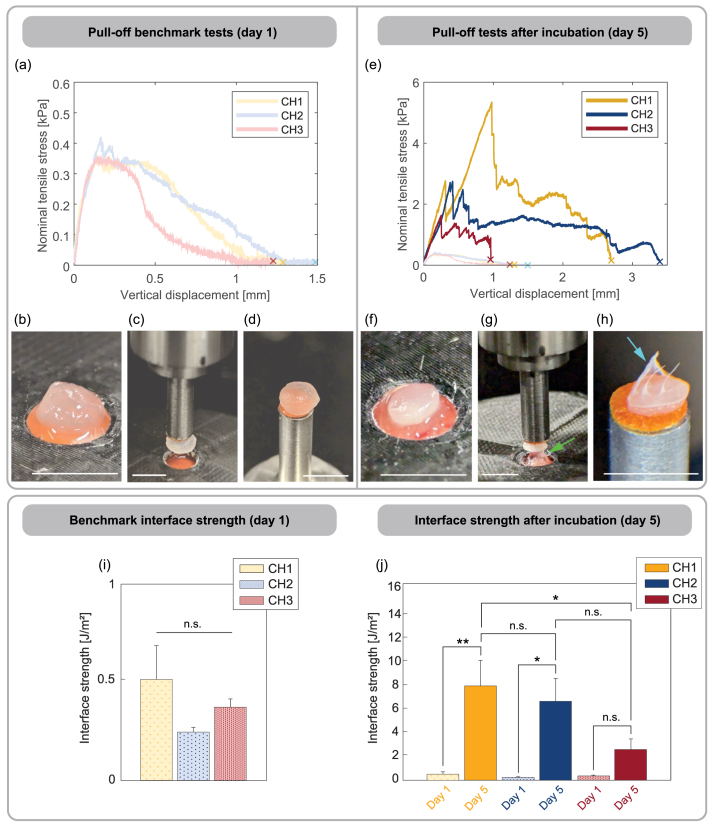
Pull-off tests and interface strength assessment in different hydrogel compositions. (a) Nominal tensile stress obtained from benchmark tests on day 1. The point of complete detachment is indicated with cross marks; (b)–(d) images show a representative hydrogel sample CH1 before, during and after pull-off. (e) Nominal tensile stress obtained from pull-off tests after 5 days of incubation, compared to day 1. The point of complete detachment is indicated with cross marks; (f)–(h) images showing a representative hydrogel sample (CH2) before, during, and after pull-off. The green arrow indicates the interface during pull-off; the cyan arrow indicates remains of the CAM after full detachment. (i) Interface strength of hydrogels on day 1. No significance (n.s.) was detected between the three hydrogel compositions CH1 (n=2), CH2 (n=3) and CH3 (n=3); (j) comparison of derived interface strength on day 1 and on day 5 for CH1 (n=6), CH2 (n=5) and CH3 (n=4), where *p<0.05, **p<0.01. Numerical data are represented as mean and standard deviation. (For interpretation of the references to color in this figure legend, the reader is referred to the web version of this article.)

Further information on biointegrability was derived from the mechanical pull-off tests of the hydrogel discs from the CAM membrane (see Fig. 3(c)). The uniaxial pull-off forces F, measured after 1 day and 5 days of incubation, were converted to nominal tensile stresses using the respective contact area of each hydrogel sample. This quantity describes the load at the interface between the hydrogel and the membrane; further dissipative processes in material behavior were not taken into account. Representative curves for each hydrogel composition, together with images of the samples before, during, and after pull-off, are shown in Fig. 5(a)–(h).

The stress curves of the benchmark tests on day 1 (Fig. 5(a)) have the typical form of axisymmetric tack tests, commonly used to test the adhesion of films and interfaces [90], [91], [92], [93]. The mechanical response is characterized by a steep, almost linear increase of the nominal stress, followed by an abrupt drop in force. As only minutes elapsed between application of the hydrogel sample on the CAM membrane and pull-off, any influence of cell infiltration on the adhesive forces that were measured can be excluded. Consequently, only the pure adhesion of the hydrogel is assessed at this stage. The nominal tensile stresses are on the order $O\left(10^{-1}\right)$  kPa, which is at the lower end of the spectrum for adhesion of similar hydrogels on soft biological tissues [94], [95], [96], [97]. The benchmark tests do not show significant qualitative or quantitative differences for the different hydrogel compositions.

Fig. 5(e) shows the nominal tensile stresses obtained from the test after 5 days of incubation. Strikingly, increased tensile stresses in the order $O\left(10^{0}\right)$  kPa to $O\left(10^{1}\right)$  kPa were observed in all pull-off tests, indicating enhanced attachment of the hydrogel discs. The displacement required for full detachment is also significantly increased, with the notable exception of the CH3 composition. Qualitatively, these results suggest an evident trend: the softer hydrogel (CH1) tends to adhere more strongly than the stiffer and denser compositions (CH2 and CH3), likely due to differences in the interface region affecting cell infiltration in the hydrogel. To substantiate these observations, we computed the interface strength and established a quantitative comparison across the different hydrogel compositions.

#### 3.2.4 Quantitative analysis reveals that ultrasoft hydrogels show enhanced interface strength

We derived the interface strength for each sample using Eq. (2) and performed a systematic statistical analysis of the corresponding mean values for pairwise comparisons among the hydrogels CH1, CH2, and CH3 on both day 1 and day 5. The interface strength results from the benchmark tests on day 1 are shown in Fig. 5(i). Comparing the means of CH1, CH2, and CH3 on day 1 revealed no significant differences, confirming the observation that the adhesive behavior of the hydrogel is independent of the composition. Additionally, any correlation with sample mass was excluded in advance (see Supplementary Material).

Fig. 5(j) shows the complete pairwise comparison between the hydrogels, including the interface strength evaluated after day 5. These results reveal a statistically significant increase in interface strength for CH1 between day 1 and day 5 (p=0.008), suggesting that CH1, the softest and most porous composition, developed substantial attachment during this period. Similarly, CH2 showed a statistically significant increase in interface strength from day 1 to day 5 (p=0.020). In contrast, the densest and stiffest hydrogel CH3 showed no significant difference in interface strength between day 1 and day 5 (p=0.073). Pairwise comparisons between the hydrogels on day 5 revealed further insights into the interface behavior. Hydrogel CH1 exhibited a significantly higher interface strength compared to CH3 on day 5 (p=0.028). While there is a trend towards a difference between CH1 and CH2 (p=0.062), and between CH2 and CH3 (p=0.079), both did not reach statistical significance.

Combining the observations from all mechanical tests with the qualitative assessment of the microstructure porosity, relevant insights can be gained. Hydrogel CH3 presents a notably denser microstructure with respect to the other compositions (Fig. 2, Fig. 4(c)). Accordingly, it also deviates significantly with respect to CH1 and CH2 in terms of characteristics and peak stresses in compression–tension experiments (Fig. 4(a)). To complete the comparison, the statistical analysis of interface strength in Fig. 5(i)–(j) clearly revealed a distinct response of the denser hydrogel composition, showing very reduced attachment and integration with respect to the softer and more porous counterparts. Additional comparison between the interface strength and the averaged peak stresses from compression–tension revealed further meaningful correlations (see Supplementary Material). Specifically, we found a strong negative correlation ($R^{2}=0.999$) between peak stresses and interface strength, suggesting that the softer materials achieve stronger attachment to the chorioallantoic membrane. These results should, however, be considered with care, as such a simplification fails to adequately represent the complexity of these porous, viscoelastic biomaterials [68], [70]. Accurate material modeling with subsequent parameter estimation is necessary to identify in detail the correlations between microstructure, stiffness, and interface strength. Given the scope of this study, this aspect was not further pursued. Here, we focused instead on gaining a deeper insight into the origin of the interface strength by analyzing histological sections of the CAM and hydrogel samples.

**Fig. 6:**
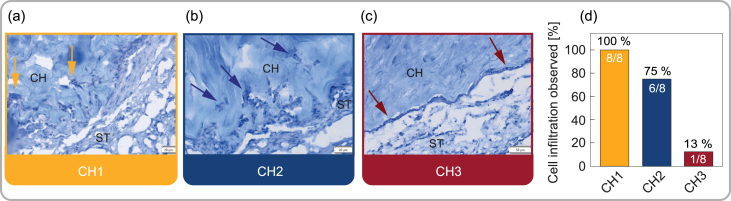
Histological assessment of the interface between hydrogel and CAM. Representative images of hydrogel–CAM interfaces, after 5 days of incubation, of (a) CH1, (b) CH2 and (c) CH3 are shown. The arrows indicate the cells at the interface of ectoderm and composite hydrogels (CH). The underlying stroma (ST) is also indicated. Samples were cryosectioned and stained with Hematoxylin and Eosin. (d) Quantitative assessment represented by observed cases of cell infiltration, with n=8 for each hydrogel composite.

#### 3.2.5 Histological examinations reveal increased cell infiltration in soft hydrogels

The histological investigation revealed distinct changes of the CAM in response to the different hydrogel compositions (Fig. 6). In specific cases, the ectoderm was characterized by the infiltration of cells into the hydrogel pores. The infiltration was qualitatively classified as either “present” or “absent” to assess the effect frequency in relation to the hydrogel composition. Specifically, cellular infiltration and the associated change in the CAM occurred more frequently in the softer hydrogels, CH1 and CH2 (see Fig. 6(a)–(c)). Fig. 6(d) shows the observed cases of cell infiltration into the hydrogels. Cell infiltration/migration was observed in 100% of CH1 samples. In comparison, 75% of the CH2 samples exhibited cell migration from the CAM. In the CH3 samples, the lowest extent of cell infiltration was observed, with only one out of eight samples (13%) showing migrated cells.

We hypothesize that the enhanced pore size in combination with lower material stiffness facilitated increased cell migration into the softer hydrogel compositions [20], [98]. As the cryo-SEM micrographs in Fig. 4(c) clearly illustrate, CH1 has a fine and loose polymer network characterized by large, interconnected pores. This distinct structure appears to be sufficiently porous and flexible, providing sufficient space for cell infiltration at the hydrogel–CAM interface. The infiltration of cells may result in mechanical bonding between hydrogel and host membrane based on morphological locking, with tightly accumulated cells at the fringes of the interface [99]. In this context, morphological locking refers to the physical anchoring created with the porous surface of the hydrogels, which can facilitate cell accumulation and thus enhance the bond strength at the interface [93]. In contrast, the denser and stiffer composition CH3 seems not to provide an appropriate environment for cells, also accounting for the lack of significant increase in interface strength compared to the benchmark test on day 1 (see Fig. 5(j)). Analogous influence of the morphology on the scaffold-membrane interface behavior has already been reported [20], [99], [100], [101].

A potential limitation of this study is the lack of investigation of hydrogel degradation under various conditions. While the materials used are known for their stability in hydrated environments over short periods of time, degradation during longer interactions could affect biointegration. Future research should address long-term stability and degradation dynamics to better understand their impact on hydrogel performance in broader applications.

## 4 Conclusion

In this work, the biointegration of soft mimicking hydrogels with chorioallantoic membrane (CAM) as host tissue was qualitatively and quantitatively assessed, by using a combination of mechanical and histological analyses. The results obtained suggest that hydrogel properties, particularly the porosity and the stiffness, play a crucial role in determining the extent of CAM changes and remodeling, and thus the biomaterial integration into biological systems.

Specifically, biomechanical assessment from pull-off tests supported by histological investigations, revealed that the hydrogel–CAM interface showed distinct cell migration, leading to morphological locking and increase in interface strength for softer and more porous hydrogels. While these results emphasize the interplay between stiffness, microstructure and integration in the chicken embryo model, it is important to note that they cannot be directly generalized to *in vivo* applications. Moreover, our study focuses on PVA-based hydrogels only; nevertheless, insights into the role of stiffness and microstructure can potentially be expanded to similar hydrogel compositions, particularly those containing synthetic polymers.

This study advances our understanding of scaffold–host interactions in dynamic biological environments, with broad implications for developing physiologically relevant CAM-grafted cell culture models. Future studies should focus on systematically investigating variations of the physical parameters of hydrogels relevant for cell infiltration, particularly porosity and permeability. In addition, a more precise determination of the immune response, morphological changes, and involved cell types requires specific antibody staining, an aspect that also deserves further investigation.

## CRediT authorship contribution statement

**Manuel P. Kainz:** Writing – review & editing, Writing – original draft, Visualization, Methodology, Investigation, Formal analysis, Data curation, Conceptualization. **Mathias Polz:** Writing – review & editing, Writing – original draft, Visualization, Methodology, Investigation, Data curation, Conceptualization. **Daniel Ziesel:** Writing – review & editing, Methodology. **Marta Nowakowska:** Investigation, Data curation. **Muammer Üçal:** Writing – review & editing, Investigation, Data curation. **Sabine Kienesberger:** Methodology, Investigation, Formal analysis. **Sophie Hasiba-Pappas:** Writing – review & editing, Conceptualization. **Raimund Winter:** Writing – review & editing, Conceptualization. **Nassim Ghaffari Tabrizi-Wizsy:** Writing – review & editing, Supervision, Methodology, Investigation. **Sarah Kager:** Methodology, Investigation. **Theresa Rienmüller:** Writing – review & editing, Supervision, Project administration, Funding acquisition, Conceptualization. **Julia Fuchs:** Writing – review & editing, Visualization, Methodology. **Michele Terzano:** Writing – review & editing, Methodology, Conceptualization. **Christian Baumgartner:** Writing – review & editing, Supervision, Conceptualization. **Gerhard A. Holzapfel:** Writing – review & editing, Supervision, Resources, Project administration, Investigation, Funding acquisition.

## Declaration of competing interest

The authors declare that they have no known competing financial interests or personal relationships that could have appeared to influence the work reported in this paper.

## Data Availability

Data will be made available on request.

## Appendix A Supplementary data

MMC S1 Further information is provided in the Supplementary Material.
