# Supplementary material for: Biointegration of soft tissue-inspired hydrogels on the chorioallantoic membrane: An experimental characterization
Source: Mater Today Bio. 2025 Jan 30;31:101508. doi: 10.1016/j.mtbio.2025.101508 (PMC11846936; doi:10.1016/j.mtbio.2025.101508)
Supplement: MMC S1 — Further information is provided in the Supplementary Material. [file mmc1.pdf]

Supplementary Material for Manuscript:
'Biointegration of soft tissue-inspired hydrogels on the chorioallantoic membrane: An experimental characterization'

M.P. Kainz, M. Polz, D. Ziesel, M. Nowakowska, M. Ucal, S. Kienesberger, S. Hasiba-Pappas, R. Winter, N.G. Tabrizi-Wizsy, S. Kager, T. Rienmüller, J. Fuchs, M. Terzano, C. Baumgartner and G.A. Holzapfel*

*Corresponding author

List of contents

1 **Collection of studies that have previously explored hydrogels in CAM assays** (supplementing 1 Introduction)

2 **Validation of sterilization procedure** (supplementing 2.1.1 Sterile hydrogel fabrication)

3 **Detailed procedure for *ex ovo* cultivation of chick embryos** (supplementing 2.2.1 Hydrogels on CAM)

4 **Statistical analysis of cytotoxicity assay** (supplementing 3.1.1 High sensitivity model shows no cytotoxic effects of the hydrogel on maturing neurons)

5 **Statistical analysis of embryo survival rate** (supplementing 3.1.2 Hydrogels have no influence on embryo survival rate)

6 **Biomechanical characterization: Effect of conditioning and hysteresis during the compression-tension tests** (supplementing 3.2.1 Mechanomimetic behavior of hydrogels can be tuned by varying the polymer concentration)

7 **Enlarged views of microscopy images** (supplementing 3.2.2 Relationship between network density and polymer concentration confirmed across hydrogels CH1 to CH3)

8 **Correlation of interface strength and sample mass** (supplementing 3.2.4 Quantitative analysis reveals that ultrasoft hydrogels show enhanced interface strength)

9 **Correlation of interface strength and compressive/tensile peak stresses** (supplementing 3.2.4 Quantitative analysis reveals that ultrasoft hydrogels show enhanced interface strength)

1 Collection of studies that have previously explored hydrogels in CAM assays (supplementing 1 Introduction)

Table 1 presents a compilation of studies that have previously investigated hydrogels in combination with CAM assays, emphasizing the main hydrogel type (polymers) used. Additionally, the specific application of hydrogels in conjunction with the CAM is discussed, underscoring the gap in the literature regarding the mechanical interface behavior of this commonly used model.

Table 1 Collection of studies describing hydrogels in combination with chorioallantoic membrane (CAM) assays.

Ref.	Title	Main hydrogel type	Use of CAM model
[1]	Vascularized nanocomposite hydrogel mechanically reinforced by polyelectrolyte-modified nanoparticles	Hyaloronic acid and chitosan	Cell invasion, vascularization, angiogenesis
[2]	Visible light-induced 3D bioprinted injectable scaffold for minimally invasive tissue regeneration	Gelatin methacrylate (GelMA) and poly(ethylene glycol) diacrylate (PEGDA)	Angiogenesis
[3]	Rational design of antimicrobial peptide conjugated graphene-silver nanoparticle loaded chitosan wound dressing	Chitosan	Angiogenesis
[4]	A bio-inspired, microchanneled hydrogel with controlled spacing of cell adhesion ligands regulates 3D spatial organization of cells and tissue	Alginate	Vascularization, angiogenesis
[5]	Dual functionalized injectable hybrid extracellular matrix hydrogel for burn wounds	Chitosan	Angiogenesis
[6]	Material characterization for wound healing potential	Protein-based human albumin hydrogel	Angiogenesis
[7]	Ionically cross-linked alginate-chitosan core-shell hydrogel beads for oral delivery of insulin	Alginate, chitosan	Angiogenesis, biocompatibility
[8]	Nanoengineered injectable hydrogels derived from layered double hydroxides and alginate for sustained release of protein therapeutics in tissue engineering applications	Alginate	Angiogenesis
[9]	The choice of biopolymer is crucial to trigger angiogenesis with vascular endothelial growth factor releasing coatings	Gelatin	Angiogenesis
[10]	Thyroxine-loaded chitosan/carboxymethyl cellulose/hydroxyapatite hydrogels enhance angiogenesis in *in-ovo* experiments	Chitosan, cellulose	Angiogenesis, vascularization

[11]	Dual independent delivery of pro-angiogenic growth factors from starPEG-heparin hydrogels	Polyethylene glycol (PEG) and heparin	Angiogenesis, vascularization
[12]	Optimizing phenol-modified hyaluronic acid for designing shape-maintaining biofabricated hydrogel scaffolds in soft tissue engineering	Hyaloronic acid, gelatin methacrylate (GelMA)	Angiogenesis, biocompatibility
[13]	In vitro Evaluation of ASCs and HUVECs Co-cultures in 3D Biodegradable Hydrogels on Neurite Outgrowth and Vascular Organization	Gellan gum	Angiogenesis, vascularization
[14]	Preclinical therapeutics ex ovo quail eggs as a biomimetic automation-ready xenograft platform	Matrigel (cover only)	Drug administration
[15]	Two-tier hydrogel degradation to boost endothelial cell morphogenesis	Polyethylene glycol (PEG) and heparin	Angiogenesis, vascularization
[16]	Curcumin cross-linked collagen aerogels with controlled anti-proteolytic and pro-angiogenic efficacy	Collagen (aerogel)	Angiogenesis
[17]	Polydeoxyribonucleotide-delivering therapeutic hydrogel for diabetic wound healing	Alginate	Angiogenesis
[18]	Visualization of micro-agents and surroundings by real-time multicolor fluorescence microscopy	Fibrin	Vascularization, micro agent detection
[19]	Assessment of flow within developing chicken vasculature and biofabricated vascularized tissues using multimodal imaging techniques	Gelatin, fibrin	Vascularization, blood floc, cell movement
[20]	In vitro and in ovo impact of the ionic dissolution products of boron-doped bioactive silicate glasses on cell viability, osteogenesis and angiogenesis	Cultrex BME Type 3	Vascularization
[21]	Development of a highly porous bioscaffold by the combination of bubble entrapping and freezing-thawing techniques to fabricate hyaluronic acid/gelatin tri-layer wound dressing	Hyaloronic acid, gelatin	Angiogenesis

2 Validation of sterilization procedure (supplementing 2.1.1 Sterile hydrogel fabrication)

UV-absorption was measured using a fiber optic spectrometer (Avantes AvaSpec-2048x14-USB2, 200-1160 nm) to assess the penetration of UV radiation through the hydrogel for sterilization (Figure 1). Thawed hydrogels were cut into 2 mm slices using a spring steel blade and then punched out using a biopsy punch (5 mm diameter). The spectral analysis was first conducted with blank UV-light in a laminar flow hood (HMC Europe, Biosafety Cabinet BSC-700II-1). Next, the samples were placed onto the spectrometer probe to determine the sample's UV absorbance. The absorption spectrum was normalized to the blank value. Absorbance at the peak value of the UV lamp at 436 nm was 38%.

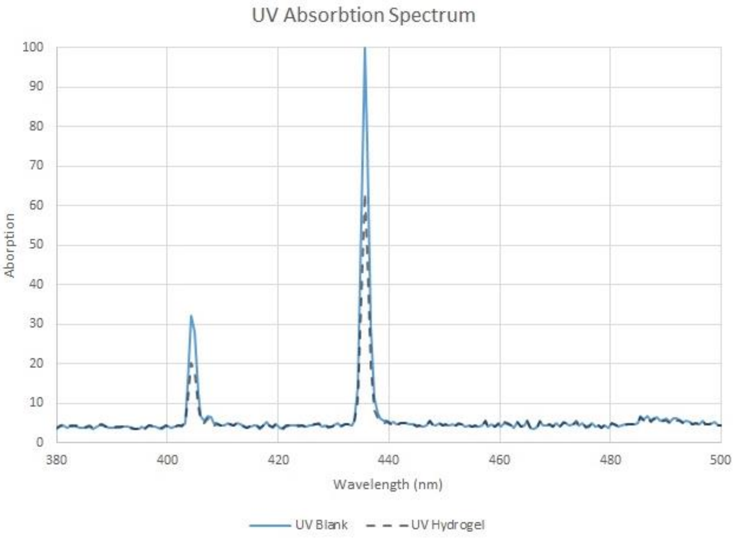

Figure 1: Normalized absorption spectrum of 2 mm thick hydrogel material (CH2). The spectrum is normalized to the blank UV spectrum of the UV source.

CH2 hydrogel bulk material and its precursor materials were analyzed for microbial contamination before and after UV sterilization at the Institute of Molecular Biosciences, University of Graz (Figure 2). Pellets of Phytagel (PHY) and polyvinyl alcohol (PVA), both individually and as a combined composite hydrogel after freezing and thawing were tested for contaminants before and after 10 min of exposure to UV light. Subsequently, pellets and hydrogel samples were subjected to microbial testing in nutrient rich medium under controlled conditions. Both precursor materials, in their raw pellet form, were separately submerged into 10 ml tryptic soy broth (TSB) medium within 50 ml Greiner tubes. Hydrogels, prepared by cutting them into small pieces of 2 mg, were placed in 75 ml TSB. All samples were incubated in an aerobic orbital incubator at 37 °C and 180 rpm.

After 48 h, contamination was observed in both pure PHY and PHY-PVA composite hydrogels that were not sterilized, as well as in samples that were UV-sterilized for 10 min. Blood agar was employed to identify contamination, which showed the presence of bacterial species after 24 h of incubation (Figure 2). The contamination became prominently evident after an additional 24 h of incubation on the blood agar plate. Identifying the PHY pellets as potential contamination sources, UV sterilization time was increased to 15 min while stirring the pellets constantly. Subsequent incubation in TSB medium as previously described showed no contamination after 14 days. The hydrogel also showed no contamination after incubation for

14 days at 37 °C and 180 rpm, demonstrating that UV sterilization with a total exposure time of 15 min is sufficient to sterilize the samples. All contamination tests were conducted in quadruplicates for each scenario (UV vs. no UV, raw pellet vs. hydrogel) to ensure reliability. The tests were repeated for every produced batch of hydrogel during the course of the experiments to ensure consistent quality control. Absorption measurements showed no significant differences between different compositions. UV sterilization did not visibly alter the microstructure of the hydrogel.

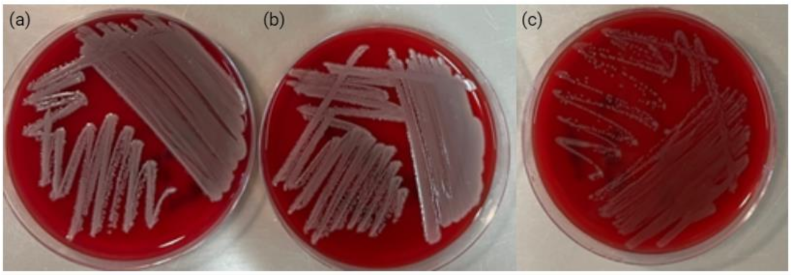

Figure 2: Blood agar plate showing bacterial colonies from a microbial burden test in (a) one out of four Phytagel samples without being exposed to UV, (b) in one out of four Phytagel samples after 10 min UV exposure, (c) in one out of four CH2 hydrogel samples without being exposed to UV. Various bacterial species are visibly growing after 48 h of incubation, indicating the microbial load present in the sample when not sterilized using UV for at least 15 min.

3 Detailed procedure for *ex ovo* cultivation of chick embryos (supplementing 2.2.1 Hydrogels on CAM)

Fertilized white leghorn eggs were washed with lukewarm water, disinfected with 75% spray alcohol, and then incubated horizontally for 72 h in an incubator with adjustable rotation at 37.7 °C and a humidity level of 75%. Rotating the eggs occasionally prevents the embryos from sticking to the shell. After incubation, the eggs were removed and carefully disinfected on the underside of the shell by dipping them into 75% ethanol before cracking them. An incision was made along the disinfected side of the egg along the equatorial line using a rotary tool with a circular saw blade at 11 000 rpm. A spacer avoided contact of the blade and the egg white and minimizes possible contamination. The developed embryo was then transferred to a weighing boat that has been washed in 75% ethanol and sterilized under UV radiation. Square Petri dishes with a custom-made air inlet were used to cover the *ex ovo* assay to avoid drying of the CAM. They were then again incubated at 37.7 °C and 75% humidity for the following experiments.

4 Statistical analysis of cytotoxicity assay (supplementing 3.1.1 High sensitivity model shows no cytotoxic effects of the hydrogel on maturing neurons)

All results shown in Figure 3 were obtained on day *in vitro* (DIV) 7. LDH release (%) was derived using Eq. 1 (main manuscrupt). The median of the control group was 19.2 with an IQR of 6.33, compared to the median of the 24 h indirect contact test of 25.3 and IQR of 9.76.

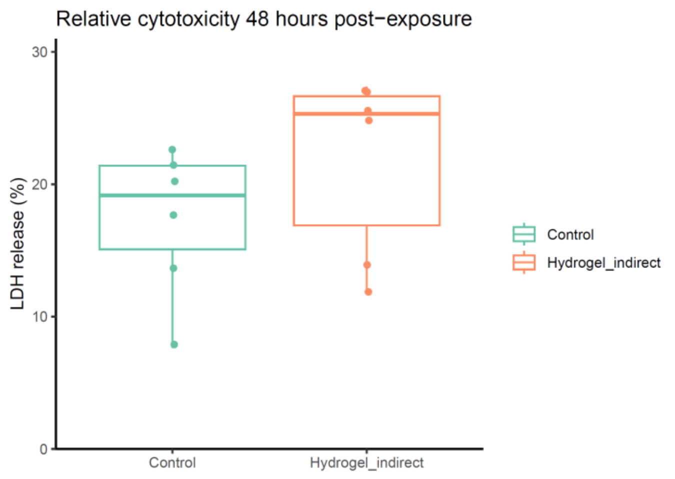

Figure 3: Results from relative cytotoxicity assessment show no significant changes of the indirect contact test (n = 6) compared to a negative control group (n = 6) on DIV7.

5 Statistical analysis of embryo survival rate (supplementing 3.1.2 Hydrogels have no influence on embryo survival rate)

A Fisher's exact test was used to compare the survival rates of chicken embryos subjected to the material with those in a negative control group (Figure 4). The quantitative comparison yielded a p-value of 1.0, indicating no statistically significant difference in survival rates between the two groups. These results suggest that the presence of the hydrogel on the CAM did not have a significantly adverse impact on embryo survival.

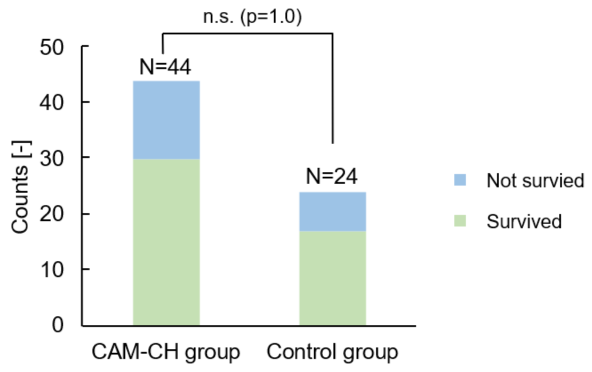

Figure 4: Survival rate of chick embryos after 14 days. The hydrogels were applied on day 9 and kept for 5 days of incubation until day 14. A total of N = 44 CAM assays were used for direct contact with the hydrogel, resulting in a survival rate of 68.2% (n = 30). The control group consisted of N = 24 assays, with a survival rate of 70.8% (n = 17).

6 Biomechanical characterization: Effect of conditioning and hysteresis during the compression-tension tests (supplementing 3.2.1 Mechanomimetic behavior of hydrogels can be tuned by varying the polymer concentration)

Cyclic compression-tension tests were performed on n = 10 (CH1), n = 6 (CH2) and n = 10 (CH3) samples. Figure 5 shows representative data for each composition. The hydrogels exhibit a (pre-) conditioning effect after the first loading, with consistent and reproducible stress-stretch behavior observed across all samples in the subsequent second to fourth cycles.

Consequently, the last cycle was used for comparative analysis of the different hydrogels in this study. Peak stresses were found to increase with increasing polymer concentration. Additionally, the enclosed area between compression and tensile stress cycles decreased with higher polymer concentration, indicating lower energy dissipation during cyclic loading. This phenomenon could be attributed to the varying water content in the composite hydrogels. CH1, which has the lowest polymer concentration and highest water content, showed the highest dissipated energy, potentially connected to the outflow of pore fluid during testing. Keeping the same loading conditions across different polymeric concentrations, the increased water content flows through the porous polymer structure, likely contributing to the higher energy dissipation observed.

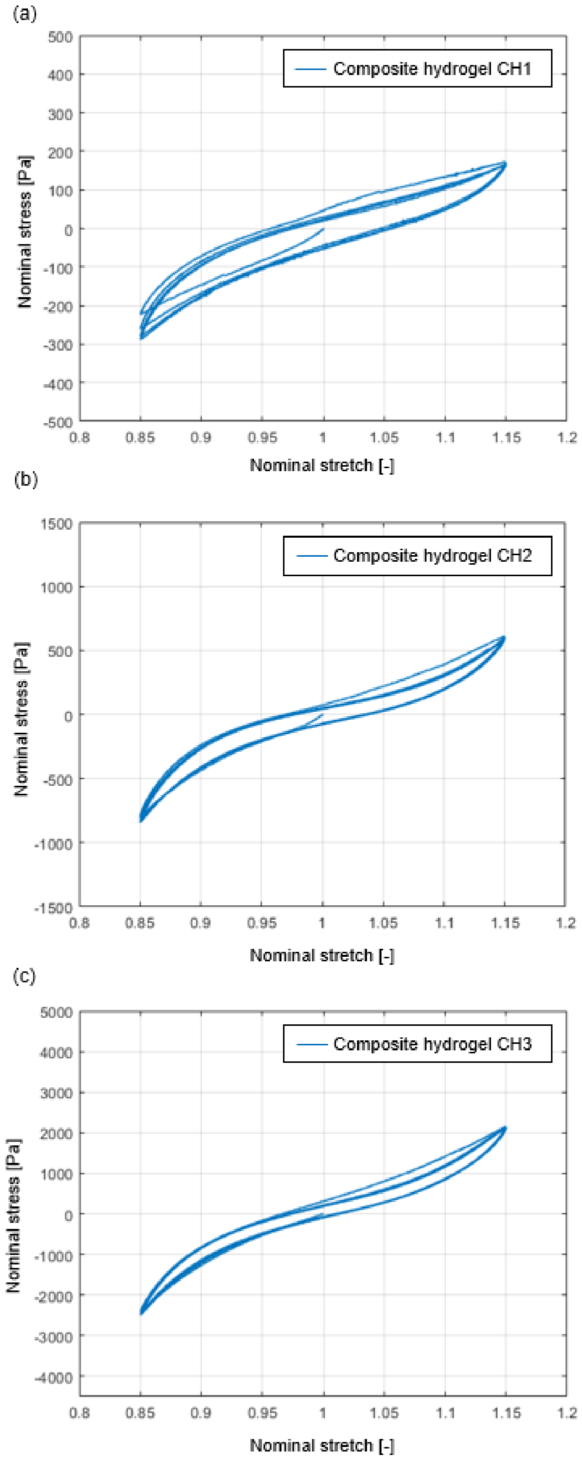

Figure 5: Representative datasets from cyclic uniaxial compression-tension testing for (a) composite hydrogel CH1, (b) CH2 and (c) CH3.

7 **Enlarged views of microscopy images** (supplementing 3.2.2 Relationship between network density and polymer concentration confirmed across hydrogels CH1 to CH3)

The images presented in Figures 6-8 show the magnified microscopy images of the composite hydrogels CH1, CH2 and CH3.

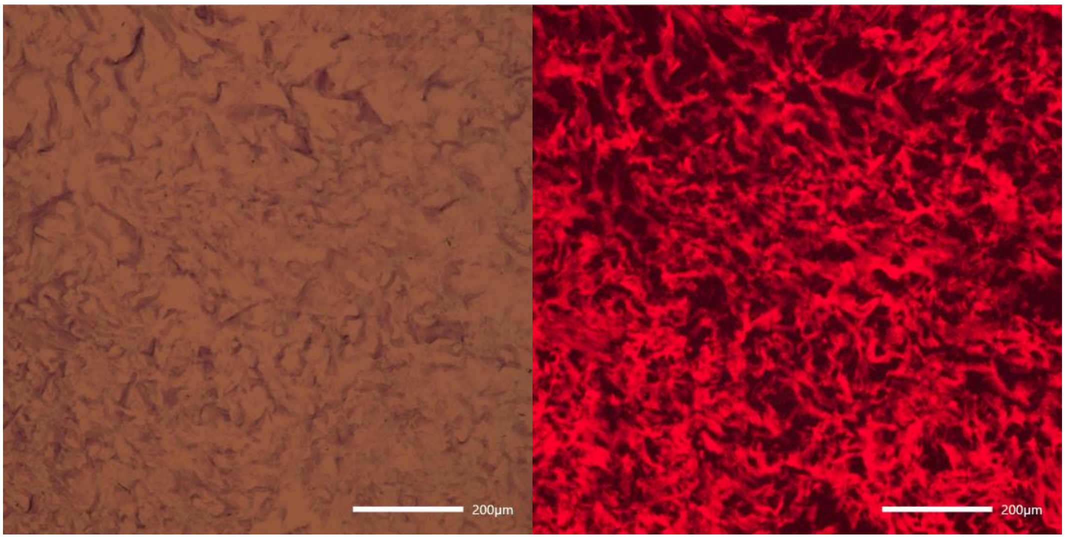

Figure 5: Original images of the hydrogel structure of the composition CH1. Cryoslices 5 µm in thickness were stained with Thionin and observed in the visible light spectrum (left) and with fluorescence microscopy excited at 470 nm (right).

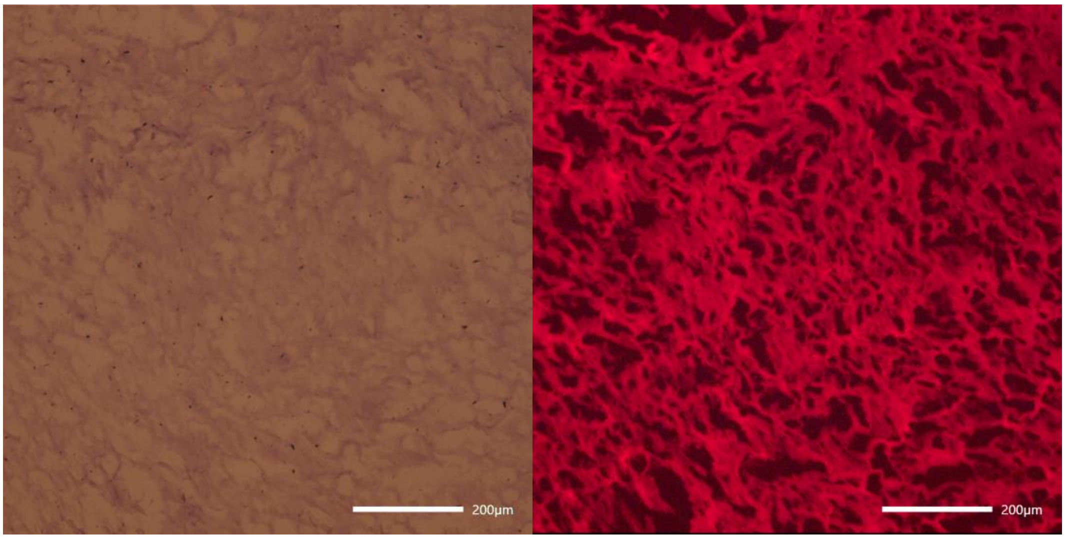

Figure 6: Original images of the hydrogel structure of the composition CH2. Cryoslices 5 µm in thickness were stained with Thionin and observed in the visible light spectrum (left) and with fluorescence microscopy excited at 470 nm (right).

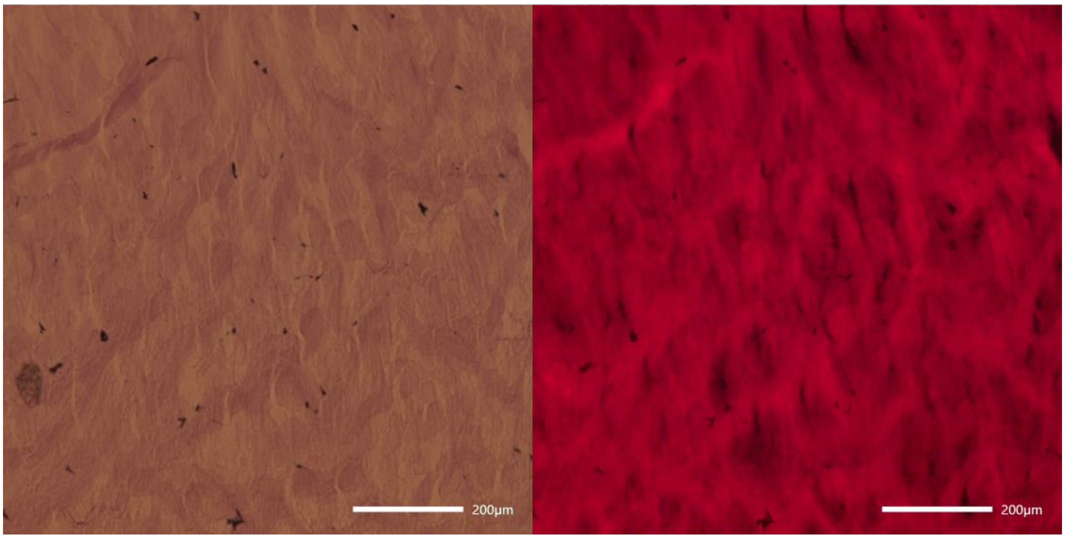

Figure 7: Original images of the hydrogel structure of the composition CH3. Cryoslices 5 µm in thickness were stained with Thionin and observed in the visible light spectrum (left) and with fluorescence microscopy excited at 470 nm (right).

8 Correlation of interface strength and sample mass (supplementing 3.2.4 Quantitative analysis reveals that ultrasoft hydrogels show enhanced interface strength)

The relationship between sample mass and interface strength is shown in the scatter plot in Figure 9. A linear regression fitted to the data yields an R^2 value of 0.002. The very low R^2 value suggests that there is no significant correlation between sample mass and interface strength. The negative slope also indicates a very weak, almost negligible inverse relationship. It can be concluded that the mass of the hydrogel samples does not have a substantial impact on the attachment, further highlighting that other factors likely play a more important role in determining the interface strength in the composite hydrogels.

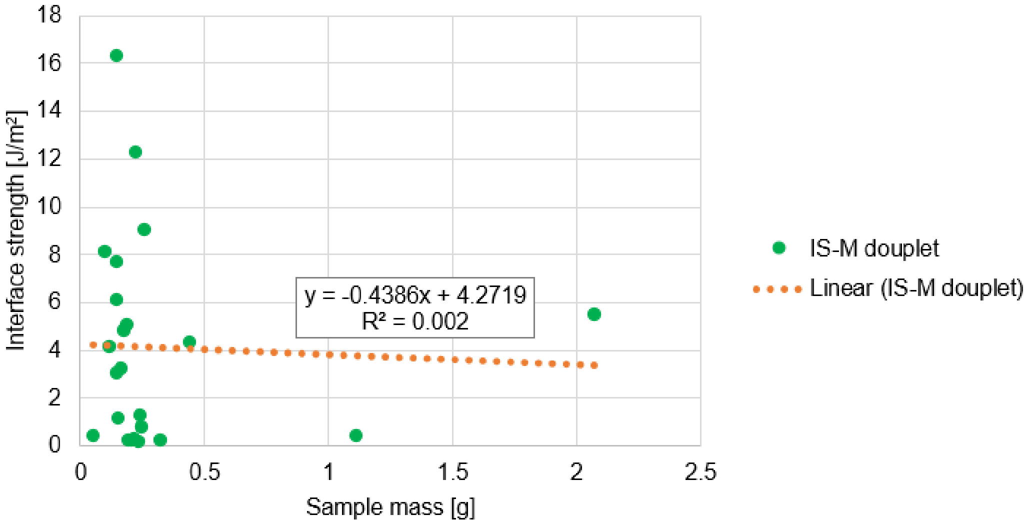

Figure 8: Scatter plot showing the distribution of the interface strength (IS) depending on the sample mass (M) for N = 24 hydrogel samples (benchmark and after incubation): n = 8 for CH1, n = 8 for CH2 and n = 7 for CH3.

9 Correlation of interface strength and compressive/tensile peak stresses (supplementing 3.2.4 Quantitative analysis reveals that ultrasoft hydrogels show enhanced interface strength)

The experimentally derived values for interface strength were plotted over the compressive and tensile peak stresses at 15 % stretch (Figure 10). The data demonstrate a strong inverse correlation between interface strength and peak stresses, both in compression (R^2 = 0.999) and tension (R^2 = 0.985). As the peak stress increases, indicating increased stiffness, the interface strength significantly decreases. This suggests that softer hydrogels, such as CH1, which exhibit lower compressive and tensile peak stresses, achieve a stronger attachment to the chorioallantoic membrane compared to stiffer hydrogels like CH3.

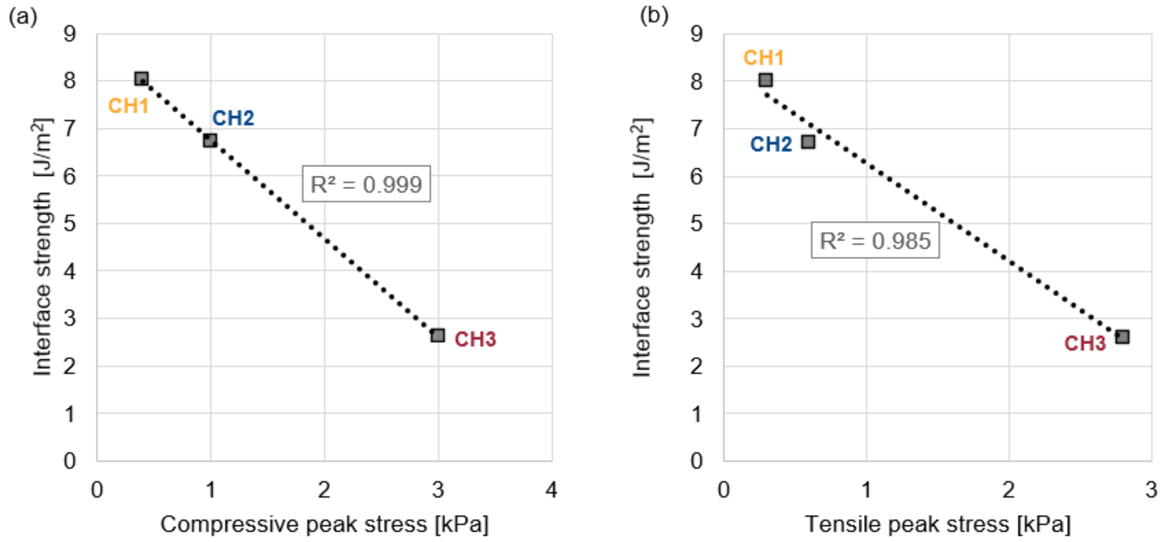

Figure 9: Correlation of interface strength: (a) compressive and (b) tensile peak stress. The stress measures refer to the nominal stress based on the cross section in the undeformed state. The dashed lines show linear regressions using the interface strength-stress pairs for each composite hydrogel.

References

[1] Zhang, Q., Pei, Q., Yang, J., Guo, S., Yang, A., Qian, Y., Li, C., Feng, Q., Lv, H., Zhou, X., He, C., Vascularized nanocomposite hydrogel mechanically reinforced by polyelectrolyte-modified nanoparticles, *J. Mater. Chem. B* 10 (2022) 5439–5453. doi:10.1039/D2TB00735E.

[2] Tilton, M., Camilleri, E. T., Astudillo Potes, M. D., Gaihre, B., Liu, X., Lucien, F., Elder, B. D., Lu, L. Visible light-induced 3D bioprinted injectable scaffold for minimally invasive tissue regeneration. *Biomater. Adv.*, 153 (2023), 213539. doi:10.1016/j.bioadv.2023.213539.

[3] Choudhary, P., Ramalingam, B., Das, S. K. Rational design of antimicrobial peptide conjugated graphene-silver nanoparticle loaded chitosan wound dressing. *Int. J. Biol. Macromol.*, 246 (2023) 125347. doi:10.1016/j.ijbiomac.2023.125347.

[4] Lee, M. K., Rich, M. H., Lee, J., Kong, H. A bio-inspired, microchanneled hydrogel with controlled spacing of cell adhesion ligands regulates 3D spatial organization of cells and tissue. *Biomater.*, 58 (2015), 26–34. doi:10.1016/j.biomaterials.2015.04.008.

[5] Bankoti, K., Rameshbabu, A. P., Datta, S., Goswami, P., Roy, M., Das, D., Ghosh, S. K., Das, A. K., Mitra, A., Pal, S., Maulik, D., Su, B., Ghosh, P., Basu, B., Dhara, S. Dual functionalized injectable hybrid extracellular matrix hydrogel for burn wounds. *Biomacromolecules*, 22 (2021), 514–533. doi:10.1021/acs.biomac.0c01400.

[6] Sharifi, S., Saei, A. A., Gharibi, H., Mahmoud, N. N., Harkins, S., Dararatana, N., Lisabeth, E. M., Serpooshan, V., Végvári, Á., Moore, A., Mahmoudi, M. Mass spectrometry, structural analysis, and anti-inflammatory properties of photo-cross-linked human albumin hydrogels. *ACS Appl. Bio Mater.* 5 (2022), 2643–2663. doi:10.1021/acsabm.2c00109.

[7] Phan, V. H. G., Mathiyalagan, R., Nguyen, M. T., Tran, T. T., Murugesan, M., Ho, T. N., Huong, H., Yang, D. C., Li, Y., Thambi, T. Ionically cross-linked alginate-chitosan core-shell hydrogel beads for oral delivery of insulin. *Int. J. Biol. Macromol.* 222 (2022), 262–271. doi:10.1016/j.ijbiomac.2022.10.174.

[8] Phan, V. H. G., Duong, H. S., Le, Q. G. T., Janarthanan, G., Vijayavenkataraman, S., Nguyen, H. N. H., Nguyen, B. P. T., Manivasagan, P., Jang, E. S., Li, Y., Thambi, T. Nanoengineered injectable hydrogels derived from layered double hydroxides and alginate for sustained release of protein therapeutics in tissue engineering applications. *J. Nanobiotechnol.* 21 (2023), 405. doi:10.1186/s12951-023-02160-2.

[9] Claaßen, C., Dannecker, M., Grübel, J., Kotzampasi, M. E., Tovar, G. E. M., Stanzel, B. V., Borchers, K. The choice of biopolymer is crucial to trigger angiogenesis with vascular endothelial growth factor releasing coatings. *J. Mater. Sci. Mater. Med.* 31 (2020), 93. doi:10.1007/s10856-020-06424-3.

[10] Malik, M. H., Shahzadi, L., Batool, R., Safi, S. Z., Khan, A. S., Khan, A. F., Chaudhry, A. A., Rehman, I. U., Yar, M. Thyroxine-loaded chitosan/carboxymethyl cellulose/hydroxyapatite hydrogels enhance angiogenesis in in-ovo experiments. *Int. J. Biol. Macromol.* 145 (2020), 1162-1170. doi:10.1016/j.ijbiomac.2019.12.082.

[11] Zieris, A., Chwalek, K., Prokoph, S., Levental, K. R., Welzel, P. B., Freudenberg, U., Werner, C. Dual independent delivery of pro-angiogenic growth factors from starPEG-heparin hydrogels. *J. Control. Release* 156 (2011), 28-36. doi:10.1016/j.jconrel.2011.07.008.

[12] Siminska-Stanny, J., Hachemi, F., Dodi, G., Cojocaru, F. D., Gardikiotis, I., Podstawczyk, D., Delporte, C., Jiang, G., Nie, L., Shavandi, A. Optimizing phenol-modified hyaluronic acid for designing shape-maintaining biofabricated hydrogel scaffolds in soft tissue engineering. *Int. J. Biol. Macromol.* 244 (2023), 125201. doi:10.1016/j.ijbiomac.2023.125201.

[13] Rocha, L. A., Gomes, E. D., Afonso, J. L., Granja, S., Baltazar, F., Silva, N. A., Shoichet, M. S., Sousa, R. A., Learmonth, D. A., Salgado, A. J. In vitro evaluation of ASCs and HUVECs co-cultures in 3D biodegradable hydrogels on neurite outgrowth and vascular organization. *Front. Cell Dev. Biol*, 8 (2020), 489. doi:10.3389/fcell.2020.00489.

[14] Rasmussen, S. V., Berlow, N. E., Price, L. H., Mansoor, A., Cairo, S., Rugonyi, S., Keller, C. Preclinical therapeutics ex ovo quail eggs as a biomimetic automation-ready xenograft platform. *Sci. Rep.*, 11 (2021), 23302. doi:10.1038/s41598-021-02509-3.

[15] Chwalek, K., Levental, K. R., Tsurkan, M. V., Zieris, A., Freudenberg, U., Werner, C. Two-tier hydrogel degradation to boost endothelial cell morphogenesis. *Biomater.*, 32 (2011), 9649-9657. doi:10.1016/j.biomaterials.2011.08.078.

[16] Dharunya, G., Duraipandy, N., Lakra, R., Korapatti, P. S., Jayavel, R., Kiran, M. S. Curcumin cross-linked collagen aerogels with controlled anti-proteolytic and pro-angiogenic efficacy. *Biomed. Mater.*, 11 (2016), 045011. doi:10.1088/1748-6041/11/4/045011.

[17] Shin, D. Y., Park, J.-U., Choi, M.-H., Kim, S., Kim, H.-E., Jeong, S.-H. Polydeoxyribonucleotide-delivering therapeutic hydrogel for diabetic wound healing *Sci. Rep.*, 10 (2020), 16811. doi:10.1038/s41598-020-74004-0.

[18] Kaya, M., Stein, F., Padmanaban, P., Zhang, Z., Rouwkema, J., Khalil, I. S. M., Misra, S. Visualization of micro-agents and surroundings by real-time multicolor fluorescence microscopy. *Sci. Rep.*, 12 (2022), 13375. doi:10.1038/s41598-022-17297-7.

[19] Padmanaban, P., Chizari, A., Knop, T., Zhang, J., Trikalitis, V. D., Koopman, B., Steenbergen, W., Rouwkema, J. Assessment of flow within developing chicken vasculature and biofabricated vascularized tissues using multimodal imaging techniques. *Sci. Rep.*, 11 (2021), 18251. doi:10.1038/s41598-021-97008-w.

[20] Decker, S., Arango-Ospina, M., Rehder, F., Moghaddam, A., Simon, R., Merle, C., Renkawitz, T., Boccaccini, A. R., Westhauser, F. In vitro and in ovo impact of the ionic dissolution products of boron-doped bioactive silicate glasses on cell viability, osteogenesis and angiogenesis. *Sci. Rep.*, 12 (2022), 8510. doi:10.1038/s41598-022-12430-y.

[21] Koohzad, F., Asoodeh, A. Development of a highly porous bioscaffold by the combination of bubble entrapping and freezing-thawing techniques to fabricate hyaluronic acid/gelatin tri-layer wound dressing. *Int. J. Biol. Macromol.*, 260 (2024), 129206. doi:10.1016/j.ijbiomac.2024.129206.
